# Long-term fertilization has different impacts on bacterial communities and phosphorus forms in sugarcane rhizosphere and bulk soils under low-P stress

**DOI:** 10.3389/fpls.2022.1019042

**Published:** 2022-09-23

**Authors:** Qihua Wu, Diwen Chen, Wenling Zhou, Xingxing Zhang, Junhua Ao

**Affiliations:** Institute of Nanfan & Seed Industry, Guangdong Academy of Sciences, Guangzhou, China

**Keywords:** phosphorus, bacterial community, rhizosphere soil, bulk soil, sugarcane

## Abstract

The application of phosphorus (P) fertilizer effectively improves soil P availability, but it also affects soil microbial communities. However, the responses of soil bacterial communities and P forms to long-term P fertilization, and the relationships of bacterial communities with soil P forms remain unclear in P-deficient field. In this study, the impacts of different P fertilization treatments (chemical nitrogen and potassium (NK); chemical N, P and K (NPK); and NPK plus straw (NPKS)) on the bacterial communities and P forms in sugarcane rhizosphere (RS) and bulk soils (BS) were evaluated. Compared with the NK, the NPK and NPKS treatments significantly (*P*<0.05) increased the yield and quality characters of sugarcane, especially under NPKS. Additionally, P fertilization significantly increased the available P (AP), soluble inorganic P (Pi) and retained Pi in both the RS and BS, but they significantly increased the Chao1 and Shannon index only in the BS; and almost all these indices were significantly higher in the RS than in the BS. The bacterial community compositions were also significantly altered by P fertilization, with major changes in the RS and minor changes in the BS. The bacterial genera that were enriched in the sugarcane rhizosphere mainly included *Bradyrhizobium*, *Rhodanobacter*, *Pseudolabrys*, *Conexibacter*, and *Burkholderia-Caballeronia-Paraburkholderia*, some of which potentially promote the plant growth. Compared to NK, functional groups involved in the cycling of carbon, N, and sulfur significantly increased or decreased with fertilizer P application. Moreover, the relative abundances of many bacterial species were significantly correlated with the soil P forms. In conclusion, long-term P fertilization altered bacterial structure and functions in P-deficient sugarcane soil, which could help the soil P cycling and suppling. The results provide useful information to stimulate the power of the microbes by fertilization measures to improve soil nutrients and crop production.

## Introduction

Phosphorus (P) is an essential macronutrient for plants and is also one of the most common yield-limiting factors. The total P content is large in most soils, but soil P availability is often low in agroecosystems because most of the P is present in forms that are not easily absorbed and used by plants (Yang et al., 2013). Fertilizer P input is an important means for ensuring the soil available P supply; however, P uptake by crops is usually less than 25%, and most of the P is believed to be fixed in the soil (Yan et al., 2008). Soil P exists in two major groups: inorganic P (Pi) and organic P (Po). Both Pi and Po have a variety of forms that differ in availability, and these P forms can be interconverted (Hou et al., 2016). The cycling of P can be affected by several complex soil properties, such as physical and chemical properties (Gama-Rodrigues et al., 2014), and biological processes, such as the activity of microorganisms and plant roots (Xu et al., 2022). Given the worldwide P limitations, an improved understanding of the factors affecting the soil P cycle and availability is needed for P management in agriculture.

To sustain crop yields in agroecosystems, the mineralization and mobilization of unavailable P to readily available P is a prerequisite. In addition to the role of plants, which can excrete acid phosphatase (ACP) and other enzymes, microorganisms, including bacteria and fungi, also play an important role in soil P cycling by excreting enzymes such as alkaline phosphatase and ACP (Zhang et al., 2021). In particular, phosphate-solubilizing bacteria (PSB) are of great importance because they are a special group of microorganisms that can dissolve P in soils (Krey et al., 2013; Zhang et al., 2022). Generally, the abundance of PSB or the genes involved in P metabolism is relatively high in P-limited environments due to selection pressures (Dai et al., 2018; Pastore et al., 2020; Ran et al., 2021). The mechanism for P solubilization by PSB is as follows: bacteria excrete enzymes such as phosphatases and phytases to mineralize organic P (Jones and Oburger, 2011), or they can release organic acids such as gluconic acid and citric acid to solubilize inorganic P (Richardson et al., 2009; Su et al., 2021). Furthermore, microbes can accelerate the recycling of nutrients in soils by decomposing organic matter such as plant leaves and roots (Maranguit et al., 2017). For instance, soils with high contents of organic matter usually exhibit high levels of P availability, which may be related to their higher biological activity (Zhang et al., 2021). Additionally, rock phosphate fertilizer combined with microbial inoculation is common in agriculture and can also affect the P status and P cycling in soils (Chauhan and Bagyaraj, 2015). Previous studies have indicated that the addition of *Pseudoalteromonas* sp. can increase the bioavailability of soil P (Liu et al., 2021) and that the inoculation of *Bacillus subtilis* with a reduction in phosphate fertilization can increase the total P accumulation in sugarcane (Rosa et al., 2020). Above all, changes in the abundances and compositions of the soil bacterial community can influence P availability and cycling in soils. Despite its important role in agroecosystems, knowledge of the relationship between the bacterial community and P forms is still limited to highly weathered soils.

In agricultural practices, fertilization regimes have been found to influence bacterial communities and functions in different soil types (Fraser et al., 2015; Dai et al., 2018; Widdig et al., 2020). Mineral P fertilizer application reduced the bacterial diversity and changed the total bacterial community and the *phoD*-harbouring bacterial community in wheat rhizospheric soil (Liu et al., 2020) and reduced the abundance of enzymes related to P cycling in paddy soils (Samaddar et al., 2018). The combined application of inorganic fertilizers with organic fertilizers such as straw or manure increased bacterial richness (Jin et al., 2021) and obviously changed the microbial community structure (Wei et al., 2017). For example, compared with chemical fertilizer, straw return combined with chemical fertilizer increased the soil bacterial Chao1 and Shannon indices and increased the abundances of Proteobacteria and Chloroflexi (Jin et al., 2021). Shifts in the diversity and composition of a bacterial community can affect the role of the bacteria in soil P cycling (Samaddar et al., 2018). Numerous studies have shown that bacterial communities can be strongly affected by fertilization regimes; however, most of these studies focused only on bulk soils (Beauregard et al., 2010; Dai et al., 2018). The diversity indices and compositions of the bacterial community in rhizosphere soils are different from those in bulk soils due to direct or indirect contact of plant roots with soil (Pang et al., 2021; Ran et al., 2021). However, information on the differences in bacterial species richness and community composition between rhizosphere and bulk soils under different fertilization treatments is scarce.

Sugarcane (*Saccharum officinarum* L.) is an important sugar crop with large biomass, a long growth season and high nutrient requirements. In China, sugarcane is mainly cultivated in red and laterite soils, which usually have low organic carbon and P availability (Su et al., 2021). Moreover, long-term continuous chemical fertilizer application has led to soil acidification and changes in the microbial community structure (Mthimkhulu et al., 2016). These factors have severely restricted the sustainable development of the sugarcane industry. To develop high-efficiency ecological agriculture, crop straw return to fields has been vigorously promoted by the Chinese government (Jiang et al., 2020). During the planting and harvesting of sugarcane, many leftovers, such as roots, stems and leaves, are produced; these plant tissues contain a large amount of carbon and other nutrients. Thus, returning the leftovers to fields is especially necessary to improve soil nutrients and increase sugarcane yield. This study collected bulk soils and rhizosphere soils from an established sugarcane field trial with low available P content under three different fertilization treatments (chemical nitrogen and potassium (NK); chemical nitrogen, phosphorus and potassium (NPK); and NPK plus straw (NPKS, the straw in this study includes the leaves and tails of sugarcane)) and analysed edaphic factors, soil P forms and soil bacterial communities. The aim was to (1) identify the differences in soil P forms, soil bacterial diversity and community composition in the bulk and rhizosphere soils after long-term fertilization and (2) explore the correlation between soil bacterial and P forms. We hypothesized that P application can increase not only the contents of P forms but also the bacterial richness and diversity and that changes in rhizosphere soils (RS) are more obvious than those in bulk soils (BS); furthermore, variation in bacterial structure can affect the transformation of soil P forms. The purpose of this study was to seek better fertilization practices under low−P stress, which could help achieve a more sustainable sugarcane system in China.

## Materials and methods

### Site description and experimental design

A long-term field experiment (established in 2013) was conducted at the Wengyuan Sugarcane Base (E 113.94°, N 24.28°), Shaoguan city, Guangdong Province, China. The area has a subtropical moist monsoon climate, with an annual average temperature of 20.2°C and annual average precipitation of 1900 mm. The soil type is classified as red earth according to Chinese soil taxonomy and as a Eutric Cambisol according to the Food and Agriculture Organization (FAO) soil taxonomy. At the beginning of the experiment, the soil physicochemical properties were as follows: pH, 5.21; SOM, 19.52 g kg^-1^; total P (TP), 0.40 g kg^-1^; available N (AN), 54.3 mg kg^-1^; available P (AP), 4.9 mg kg^-1^; and available potassium (AK), 41.6 mg kg^-1^.

The field experiment was set up in 2013, and it was arranged in a randomized block design with three replicate plots. The area of each plot was 240 m^2^ (24 m × 10 m). In this study, the following three treatments were assessed: (1) N and K (NK); (2) N, P and K (NPK), and (3) NPK plus straw (NPKS). N, P and K were applied as urea, calcium superphosphate, and potassium chloride, respectively. The same amount of chemical N, P and K fertilizer was applied in each treatment, and the applications consisted of 400 kg N ha^-1^, 210 kg P_2_O_5_ ha^-1^ and 270 kg K_2_O ha^-1^. In the NPKS treatment, the entire quantity of sugarcane straw (the straw in this study includes the leaves and tails of sugarcane) was incorporated into the soil, approximately 7.5 Mg hm^-2^ year^-1^. The contents of N, P, and K in the sugarcane straw were 0.9%, 0.2%, and 1.5%, respectively, so 67.5 kg N hm^-2^, 15 kg P hm^-2^, and 112.5 kg K hm^-2^ were return to the soil every year. Thus, the NPKS treatments received greater amounts of N, P and K than the NK and NPK treatments because the added straw. Sugarcane (*S. officinarum* L.) was the only crop grown each year, and it was sown in March and harvested in January of the following year. Herbicides were used to control weeds, and insecticides or chemicals were used to prevent pests and other diseases during sugarcane growth. The crops were harvested manually with sickles, the plants were cut close to the ground, and the harvested biomass was removed from the plots in the NK and NPK treatments. Sugarcane straw was incorporated into the soil only in the NPKS treatment.

### Sugarcane parameter estimation

Five rows of sugarcane were randomly selected from each plot, twenty sugarcane plants were selected randomly in each row, and each plant’s diameter and height were measured by tape and Vernier calliper. The average values of sugarcane plant height and stem diameter were calculated for each plot. Sucrose content was determined using the approach described by Jackson and Jayanthy (2014). Sugarcane production was measured as follows: in each plot, sugarcane was cut and harvested, the leaves and tails of sugarcane were removed (except in the NPKS treatment), and the actual harvest was measured and recorded. Then, the hectare yield was calculated.

### Soil sampling and biogeochemical analysis

On January 9, 2020, after sugarcane harvest, soil samples were collected. RS samples were collected from the roots of several sugarcane plants. Soil loosely adhered to the root surface was removed by shaking, and then the tightly bound RS was removed by brushing. Samples of RS were thoroughly mixed to obtain a composite sample. At the same time, bulk soil (BS) samples were collected between rows of sugarcane at a depth of 0-20 cm. A BS sample was collected from each of the five points (near the RS samples) in each plot. A total of 18 samples were obtained. All the samples were passed through a 2-mm sieve and divided into multiple subsamples: one subsample was air-dried for soil property analysis, and the other subsamples were immediately stored at 4°C until ACP analysis was conducted or at -40°C until soil DNA extraction was carried out.

The soil pH value was measured by a pH metre (1:2.5 soil:water). SOM was measured by a digestion method. Total P was extracted with an acid solution (H_2_SO_4_-HClO_4_), and the available P was extracted with 0.03 M NH_4_F-0.025 M HCl (Bray I test) and then measured using molybdenum blue colorimetry. Soil available N was determined using the alkaline diffusion method. Available K was extracted with 1 M NH_4_OAc and then measured by a flame photometer (Page et al., 1982).

Soil soluble Pi was extracted with 0.5 M NaHCO_3_ (pH 8.5, solution ratio 1:20). Partial extracts were digested with K_2_S_2_O_8_ and NaOH in an autoclave at 120°C to measure the total soluble P. Soluble Po was determined from the difference between total soluble P and soluble Pi. Stable Pi was extracted with 0.5 M H_2_SO_4_ (solution ratio 1:25). For the total stable P, 2.0 g of air-dried soil was calcined at 550°C in a muffle furnace for 1 h and then extracted with 50 ml of 0.5 M H_2_SO_4_. The stable Po was calculated as the difference between the two extracts. The P in all the soil extracts was quantified by the colorimetric method (Murphy and Riley, 1962).

The activities of soil extracellular ACP were measured at 37°C and ph 6.5. ACP catalysed the hydrolysis of phenyl disodium phosphate into phenol and hydrogen disodium phosphate in an acidic environment, and the activity of ACP was calculated by measuring the phenol. Phosphatase activities are expressed in nmol phenol g-1 soil h-1.

### Soil DNA extraction, sequencing and data analysis

Soil dna was extracted from 0.5 g of fresh soil samples using a powersoil dna isolation kit (mobio laboratories, carlsbad, ca, usa) following the instructions in the accompanying manual. The purity and quality of the genomic dna were checked on 0.8% agarose gels. The v3-v4 hypervariable region of the bacterial 16s rrna gene was amplified with the primers 338f (5’-actcctacgggaggcagcag-3’) and 806r (5’-ggactachvgggtwtctaat-3’) (Caporaso et al., 2012). Pcr was carried out on a mastercycler gradient (eppendorf, germany) using 25 μl reaction volumes containing 5 μl 5 × reaction buffer, 5 μl 5 × gc buffer, 2 μl dntp (2 mm), 1 μl forward primer (10 μm), 1 μl reverse primer (10 μm), 2 μl dna template, 8.75 μl ddh2o, and 0.25 μl q5 dna polymerase. The cycling parameters were 98°c for 2 min, followed by 28 cycles of 98°c for 15 s, 55°c for 30 s and 72°c for 35 s, with a final extension at 72°c for 5 min. Three pcr products per sample were pooled to mitigate reaction-level pcr biases. The pcr products were purified using a qiaquick gel extraction kit (qiagen, germany), quantified using real-time pcr, and sequenced on an illumina miseq 300 pe platform at personal biotechnology company, shanghai.

After the pcrs were carried out, the raw data were first screened and qualified and then separated using barcode sequences and trimmed with trimmomatic (version 0.36). Then, the dataset was analysed using qiime (v1.9). The sequences were clustered into operational taxonomic units (otus) by vsearch (v2.13.4) at a similarity level of 97%. The basic local alignment search tool (blast) (v2.6.0) was used to classify all the sequences into different taxonomic groups based on the silva database. Finally, based on the above analysis, alpha diversity indices (observed species, chao1, and shannon indices) were calculated by mothur software (version 1.30.1). Linear discriminant analysis (lda) coupled with effect size measurement analysis (lefse) was applied to search for statistically different biomarkers between different groups using lefse software. The relationships between soil microbial community structures and soil environmental factors were analysed by redundancy analysis (rda) using canoco software v.4.5.

### Data analysis

The ANOVA procedure in SAS (v9.2, SAS Institute Inc., USA) was used for the sugarcane parameter and soil property data analysis, and the least significant differences (LSD) test at *P*<0.05 was employed to assess the differences in the means of the three replicates of the different treatments. Pearson’s correlation coefficients were employed to test the relationships between the agronomic and quality characteristics of sugarcane, soil properties and soil bacteria.

## Results

### Agronomic and quality characteristics of sugarcane

**Figure 1:**
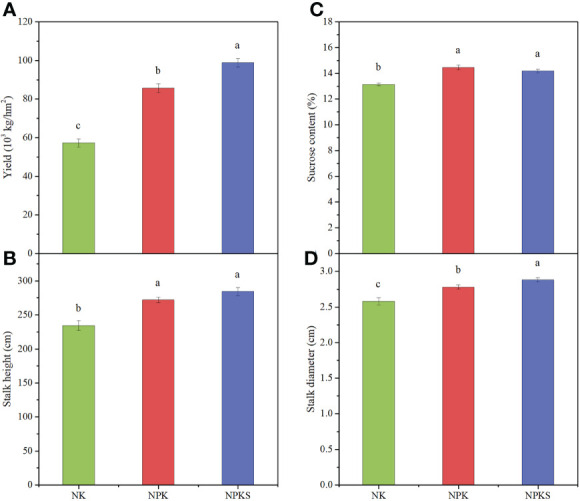
Agronomic and quality characters of sugarcane under different fertilization treatments. **(A)** Yield; **(B)** Stalk height; **(C)** Sucrose content; and **(D)** Stalk diameter. NK, chemical nitrogen and potassium; NPK, chemical nitrogen, phosphorus and potassium; NPKS, NPK plus straw. Different lowercase letters indicate significant difference among the treatments (*P* < 0.05).

The agronomic and quality characteristics of sugarcane were significantly affected by different P fertilization treatments. The sugarcane yield and stalk diameter results showed significant differences among the three fertilization treatments, following the order of NPKS>NPK>NK (**Figures 1A, D**; **Supplementary Figure 1**). The sugarcane yields in the NK treatment was 57.3×103 kg hm-2. Compared to NK, the sugarcane yields in the NPK and NPKS treatments increased by 49.5% and 72.6%, respectively. Compared with the NK treatment, the NPK and NPKS treatments significantly increased the sucrose content and stalk height (P < 0.05) (**Figures 1B, C**). Moreover, the stalk height was higher in the NPKS treatment than in the NPK treatment, whereas the sucrose content showed the reverse trend.

### Soil biogeochemical properties in the BS and RS

Compared with the NK treatment (no P application), P application significantly increased the TP, AP and pH values in both the BS and RS (*P*< 0.05) (**Table 1**). The NPKS treatment had significantly higher SOM and AK than NK, while the NPK treatment had lower AN and ACP than the NK treatment. In addition, TP, AP, AN, and AK were significantly higher in the RS than in the BS, but there was no significant difference in pH, SOM or ACP between the BS and the RS.

**Table 1:** Soil biogeochemical properties under different fertilization treatments.

Soil property	Soil	Treatment			Average
		NK	NPK	NPKS	
pH (1:2.5 H_2_O)	BS	4.90c	5.01b	5.16a	5.02A
	RS	4.67c	4.82b	5.02a	4.83A
Soil organic matter (SOM, g kg^-1^)	BS	24.5b	24.3b	29.7a	26.2A
	RS	24.7b	24.2b	33.4a	27.3A
Total P (TP, g kg^-1^)	BS	0.34c	0.40b	0.51a	0.42B
	RS	0.39c	0.49b	0.66a	0.51A
Available P (AP, mg kg^-1^)	BS	4.5c	23.9b	43.2a	23.8B
	RS	7.8c	36.4b	55.7a	33.3A
Available N (AN, mg kg^-1^)	BS	238.0a	184.0b	259.4a	227.1B
	RS	365.4a	280.8b	340.5a	328.9A
Available K (AK, mg kg^-1^)	BS	140.3b	145.0b	262.7a	182.7B
	RS	225.3b	209.3b	410.7a	281.8A
Acid phosphatase (ACP, nmol g^-1^ soil h^-1^)	BS	1576a	1185b	1505a	1421A
	RS	1397a	1269b	1342a	1336A

NK, chemical nitrogen and potassium; NPK, chemical nitrogen, phosphorus and potassium; NPKS, NPK plus straw. Different lowercase letters in the same row indicate significant differences among the treatments (P < 0.05). Different uppercase letters indicate significant differences between the bulk soil (BS) and rhizosphere (RS).

**Figure 2:**
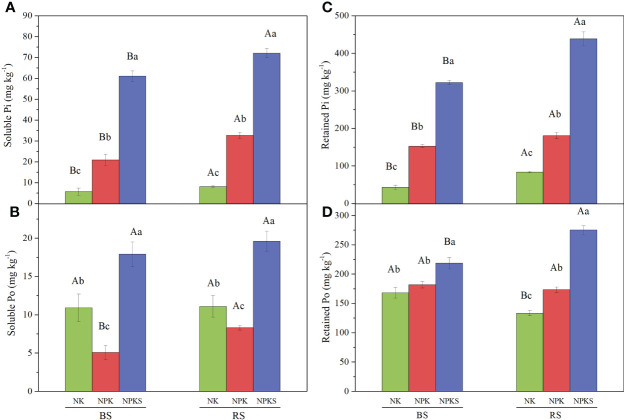
The contents of soluble P and retained P under different fertilization treatments. **(A)** Soluble Pi; **(B)** Soluble Po; **(C)** Retained Pi; and **(D)** Retained Po. NK, chemical nitrogen and potassium; NPK, chemical nitrogen, phosphorus and potassium; NPKS, NPK plus straw. Different lowercase letters indicate significant difference among the treatments (*P* < 0.05). Different uppercase letters indicate significant difference between RS (rhizosphere soil) and bulk soil (BS).

Among the soil P forms, the soluble Pi, retained Pi and retained Po contents followed the order NPKS>NPK>NK in both the BS and RS (**Figures 2A, C, D**); however, the soluble Po contents were 10.9 mg kg^-1^ in the BS and 11.1 mg kg^-1^ in the RS in the NK treatment, which were significantly higher than those in NPK treatment, with 5.1 mg kg^-1^ in the BS and 8.3 mg kg^-1^ in the RS (**Figure 2B**). Moreover, the soluble Pi, soluble Po and retained Pi contents were higher in the RS than in the BS for all three treatments, but the retained Po was higher in the BS than in the RS in the NK and NPK treatments.

### Diversity and composition of the bacterial community in the BS and RS

**Figure 3:**
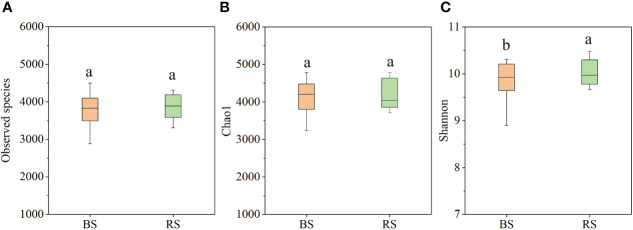
Bacterial diversity index in bulk soil (BS) and RS (rhizosphere soil). **(A)** Observed species; **(B)** Chao1; and **(C)** Shannon index. Different lowercase letters indicate significant difference between BS and RS.

Based on metagenomic analysis, the NPK and NPKS treatments significantly increased the Chao1 index and Shannon index compared with NK in the BS (*P*<0.05), whereas there were no significant differences in the observed species or Chao1 index among the three treatments in the RS (**Supplementary Table 1**). Additionally, the NK treatment significantly increased the Chao1 index and Shannon index in the RS compared with the BS, while the NPKS treatment significantly decreased the Shannon index in the RS compared with the BS. Overall, there were no significant differences in the observed species and Chao1 indices between the BS and RS (**Figures 3A, B**), while the Shannon index was significantly increased in the RS compared with that in the BS (**Figure 3C**).

**Figure 4:**
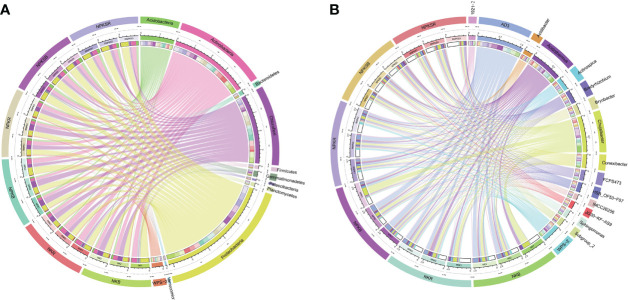
Relative abundance of bacterial community at the phylum **(A)** and genus **(B)** level under different fertilization treatments. NK, chemical nitrogen and potassium; NPK, chemical nitrogen, phosphorus and potassium; NPKS, NPK plus straw. B and R represent the BS and RS samples, respectively.

The dominant phylum in both the RS and BS was Proteobacteria, representing an average of 34.7% of all sequences, followed by Actinobacteria (23.7%), Chloroflexi (20.8%) and Acidobacteria (9.9%) (**Figure 4A**; **Supplementary Table 2**). In the RS, compared with the NK treatment, the P application treatments significantly changed the relative abundances of 5 of the 11 most abundant phyla (>1% at least one treatment), with a lower abundance of Gemmatimonadotates in the NPK treatment, lower abundances of Chloroflexi and WPS-2 in the NPKS treatment, and higher abundances of Proteobacteria and Acidobacteria in the NPKS treatment. However, in the BS, only NPKS significantly increased the relative abundances of Acidobacteria and Verrucomicrobia compared with those in the NK treatment. The 5 most abundant genera were *AD3*, *Chujaibacter*, *Acidothermus*, *WPS-2*, and *Conexibacter* in both the RS (average of 6.88%, 4.60%, 5.80%, 3.68% and 2.70%, respectively, of all sequences) and the BS (average of 9.68%, 8.51%, 5.71%, 2.89% and 2.30%, respectively) (**Figure 4B**; **Supplementary Table 3**). Among the 16 most abundant genera (>1% at least one treatment), P application significantly changed the relative abundances of 7 genera in the RS, while it changed the relative abundances of 4 genera in the BS. Compared with NK, NPKS significantly increased the abundances of *Bradyrhizobium*, *JG30-KF-AS9* and *Subgroup_2* but decreased the abundance of *Conexibacter* in both the RS and BS; NPK increased the abundances of *WPS-2* and *1921-2* only in the RS.

### Differential abundances of bacteria across fertilization treatments

**Figure 5:**
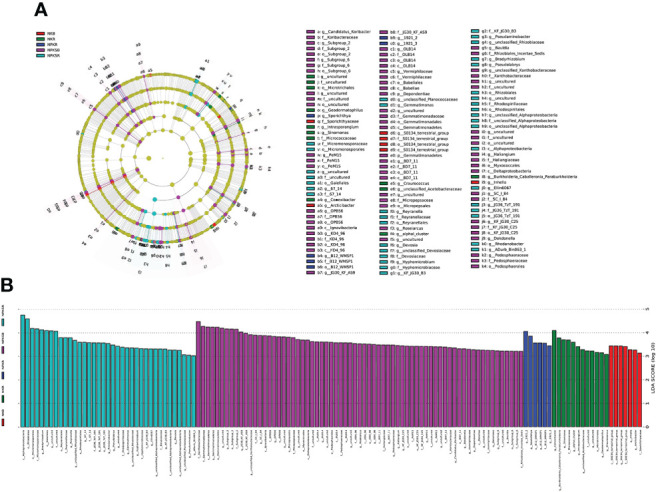
Cladograms **(A)** and biomarkes with LOA scores>3 **(B)** under different fertilization treatments confirmed by LEfSe. NK, chemical nitrogen and potassium; NPK, chemical nitrogen, phosphorus and potassium; NPKS, NPK plus straw. B and R represent the BS and RS samples, respectively.

The LEfSe tool was used to detect biomarkers from the phylum to the genus level across the different fertilization treatments. Cladograms presented different groups, and LDA scores ≥3 were confirmed by LEfSe (**Figures 5A, B**). The numbers of species enriched in the BS under NPKS (NPKSB) (69) and in the RS under NPKS (NPKSR) (37) were much higher than those in the RS under NK (NKR) (12), the BS under NK (NKB) (7) and the RS under NPK (NPKR) (6). Dependentiae and Gemmatimonadetes (phylum) were enriched in the NPKSB. Alphaproteobacteria was enriched in the NPKSR, and S0134_terrestrial_group (class) was enriched in the NKB. In the NPKSB, there were six enriched bacterial classes, of which Gemmatimonadetes and Deltaproteobacteria had the largest proportions. Furthermore, there were 24 genera enriched in the NPKSB, of which 12 belonged to Proteobacteria (6 genera), Chloroflexi (3 genera) and Acidobacteria (3 genera), and the 5 most abundant were *Subgroup_2* (3.18%), *JG30_KF_AS9* (2.66%), *SC_I_84* (1.57%), *OLB14* (0.78%), and *Roseiarcus* (0.70%). In the NPKSR, 19 genera were enriched, among which 13 belonged to Proteobacteria, including *Bradyrhizobium*, *Rhodanobacter*, *Pseudolabrys* and *Pseudaminobacter*. For the NPKR, *Sporichthya*, *B21_WMSP1*, *1921-2* and *1921-3* were enriched, and *B21_WMSP1*, *1921-2* and *1921-3* belonged to Chloroflexi. In the NKR, 9 genera were enriched, of which 4 belonged to Actinobacteria (including *Conexibacter*, *Sinomonas* and *Geodermatophilus*) and 3 belonged to Proteobacteria (*Burkholderia-Caballeronia-Paraburkholderia* and *Craurococcus*). There were 3 genera enriched in the NKB: *Arcticibacter*, *inhella*, and *S0134_terrestrial_group*.

### Association between the agronomic and quality characteristics of sugarcane, soil properties, and bacterial community

Pearson’s correlation coefficient was used to examine the relationship between soil bacterial community in RS and agronomic and quality characteristics of sugarcane (**Table 2**). At the phylum level, Acidobacteria was significantly (*P*<0.05) positively correlated with sugarcane yield, sucrose content, stalk height and stalk diameter. In contrast, Bacteroidetes had a significant negative correlation with stalk diameter. At the genus level, *Bradyrhizobium* and *Subgroup_2* showed strong and positive correlations with sugarcane yield, stalk height and stalk diameter, whereas *Conexibacter* showed negative correlations with sugarcane yield and stalk diameter (*P*<0.05).

**Table 2:** Pearson’s correlation coefficient between soil bacterial community in rhizosphere soil (RS) and agronomic and quality characteristics of sugarcane.

Phylum (relative abundance>1%)	Agronomic and quality characteristics of sugarcane				Genus (relative abundance>1%)	Agronomic and quality characteristics of sugarcane			
	Yield	Sucrose content	Stalk height	Stalk diameter		Yield	Sucrose content	Stalk height	Stalk diameter
Chloroflexi	-0.321	0.131	-0.279	-0.295	*Chujaibacter*	-0.241	-0.581	-0.270	-0.326
Proteobacteria	0.541	0.036	0.458	0.507	*Acidothermus*	-0.601*	-0.439	-0.417	-0.446
Acidobacteria	0.802**	0.599*	0.763**	0.864**	*Bradyrhizobium*	0.733*	0.427	0.734*	0.732*
Actinobacteria	-0.340	-0.513	-0.286	-0.334	*Bryobacter*	0.313	0.119	0.378	0.445
WPS-2	-0.289	0.202	-0.184	-0.232	*Subgroup_2*	0.757**	0.465	0.721*	0.792**
Planctomycetes	0.185	0.305	0.035	0.048	*JG30-KF-AS9*	0.709*	0.285	0.564	0.647
Verrucomicrobia	0.566	0.216	0.395	0.466	*Acidibacter*	0.390	0.238	0.425	0.513
Bacteroidetes	-0.464	-0.445	-0.546	-0.624*	*Actinospica*	-0.100	-0.153	-0.161	-0.185
Patescibacteria	-0.406	-0.495	-0.419	-0.528	*HSB_OF53-F07*	0.299	0.470	0.294	0.262
Firmicutes	-0.116	-0.473	-0.204	-0.197	*FCPS473*	-0.245	0.151	-0.241	-0.292
Gemmatimonadetes	0.333	-0.098	0.266	0.294	*WPS-2*	-0.289	0.202	-0.184	-0.232
					*Conexibacter*	-0.662*	-0.345	-0.506	-0.604*
					*AD3*	-0.503	-0.059	-0.427	-0.424
					*Sphingomonas*	-0.155	0.040	-0.146	-0.231
					*IMCC26256*	-0.310	0.006	-0.156	-0.247
					*1921_2*	-0.183	0.289	-0.151	-0.214

Bold values highlighted the significant relationships. *indicates P < 0.05 and ** indicates P < 0.01.

**Figure 6:**
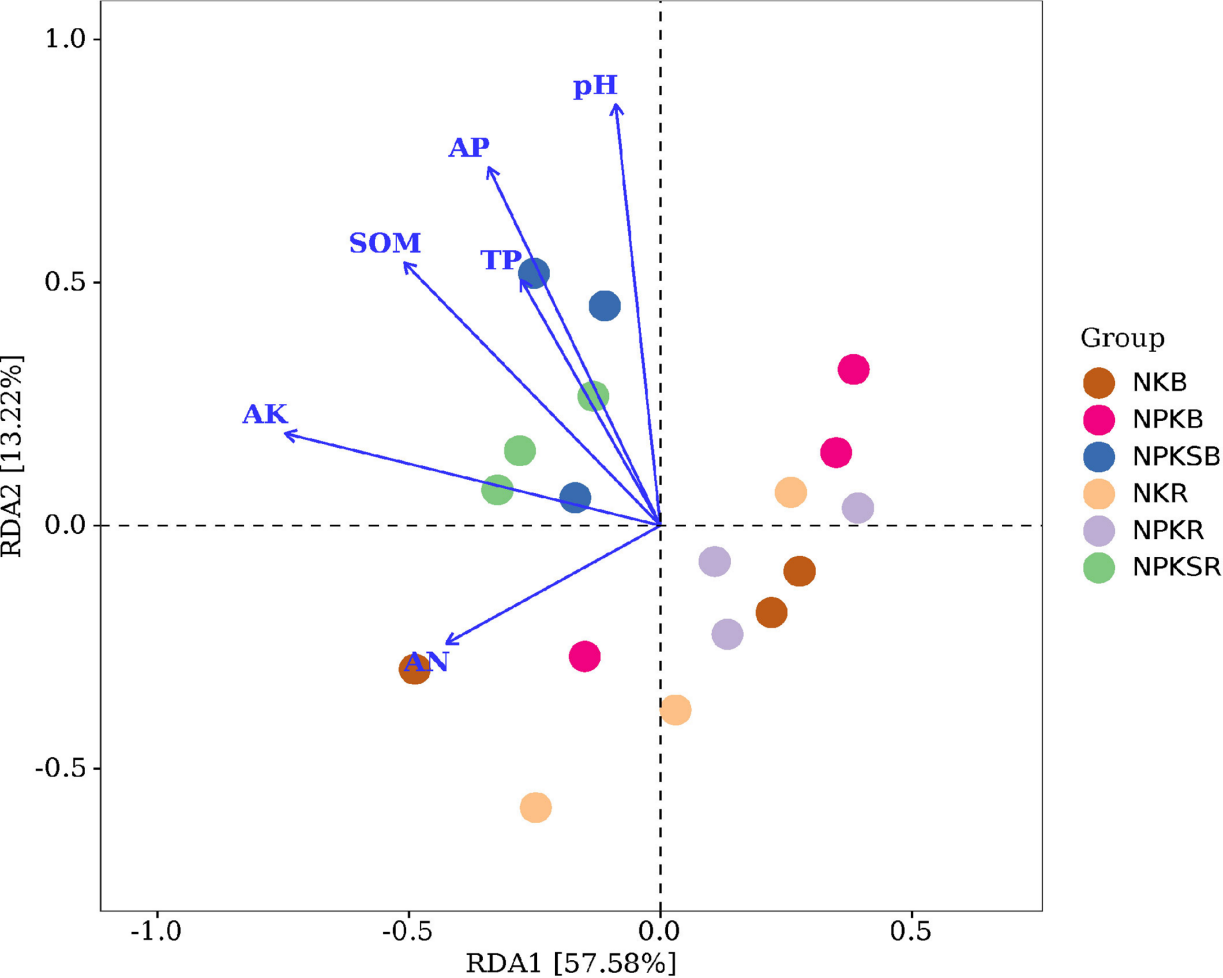
Redundancy analysis (RDA) plot revealing the relationships between bacterial communities and soil properties. NK, chemical nitrogen and potassium; NPK, chemical nitrogen, phosphorus and potassium; NPKS, NPK plus straw. B and R represent the BS and RS samples, respectively.

Redundancy analysis (RDA) was used to reveal relationships between the bacterial community and soil properties. We found that pH, SOM, TP, AP, AN and AK significantly affected the soil bacterial community (P<0.05) (**Figure 6**), indicating that these soil properties played important roles in structuring the microbial communities in both the BS and RS. Furthermore, pH, SOM AP, AN and AK significantly influenced the bacterial community in the BS (**Supplementary Figure 2A**), while pH, SOM, TP, AP and AK significantly influenced the bacterial community in the RS (**Supplementary Figure 2B**). This suggested that the influencing factors driving the soil bacterial community compositions were different between the BS and RS.

**Figure 7:**
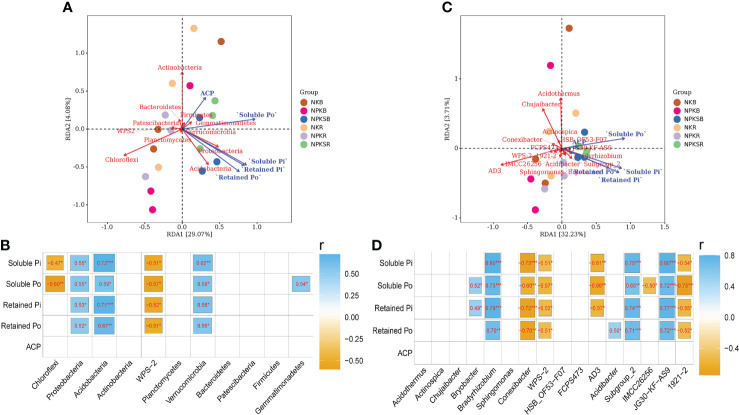
Correlation analysis for the content of soil P froms, soil acid phosphatase and the relative abundance of bacterial at the phylum **(A, B)** and genus **(C, D)** level. NK, chemical nitrogen and potassium; NPK, chemical nitrogen, phosphorus and potassium; NPKS, NPK plus straw. B and R represent the BS and RS samples, respectively. *P < 0.05, **P < 0.01, and ***P < 0.001.

The treatments with fertilizer P strongly affected the relative abundances of the main phyla and genera (>1% at least one treatment) of soil bacteria compared with the treatment without P (**Figure 4**; **Supplementary Tables 2, 3**). The relationships among soil P forms, ACP associated with these main bacterial phyla and genera were analysed (**Figure 7**). At the phylum level, Proteobacteria, Acidobacteria and Verrucomicrobia were significantly positively correlated with all four P forms, and Gemmatimonadetes had a significant positive correlation with soluble Po (*P*<0.05). In contrast, WPS-2 had a significant negative correlation with all P forms, and Chloroflexi was negatively correlated with soluble P (**Figures 7A, B**). At the genus level, *Bradyrhizobium*, *Subgroup_2* and *JG30-KF-AS9* showed strong and positive correlations with all four P forms, whereas *Conexibacter*, *WPS-2* and *1921-2* showed the opposite result (**Figures 7C, D**).

### The analysis of bacterial functions

**Figure 8:**
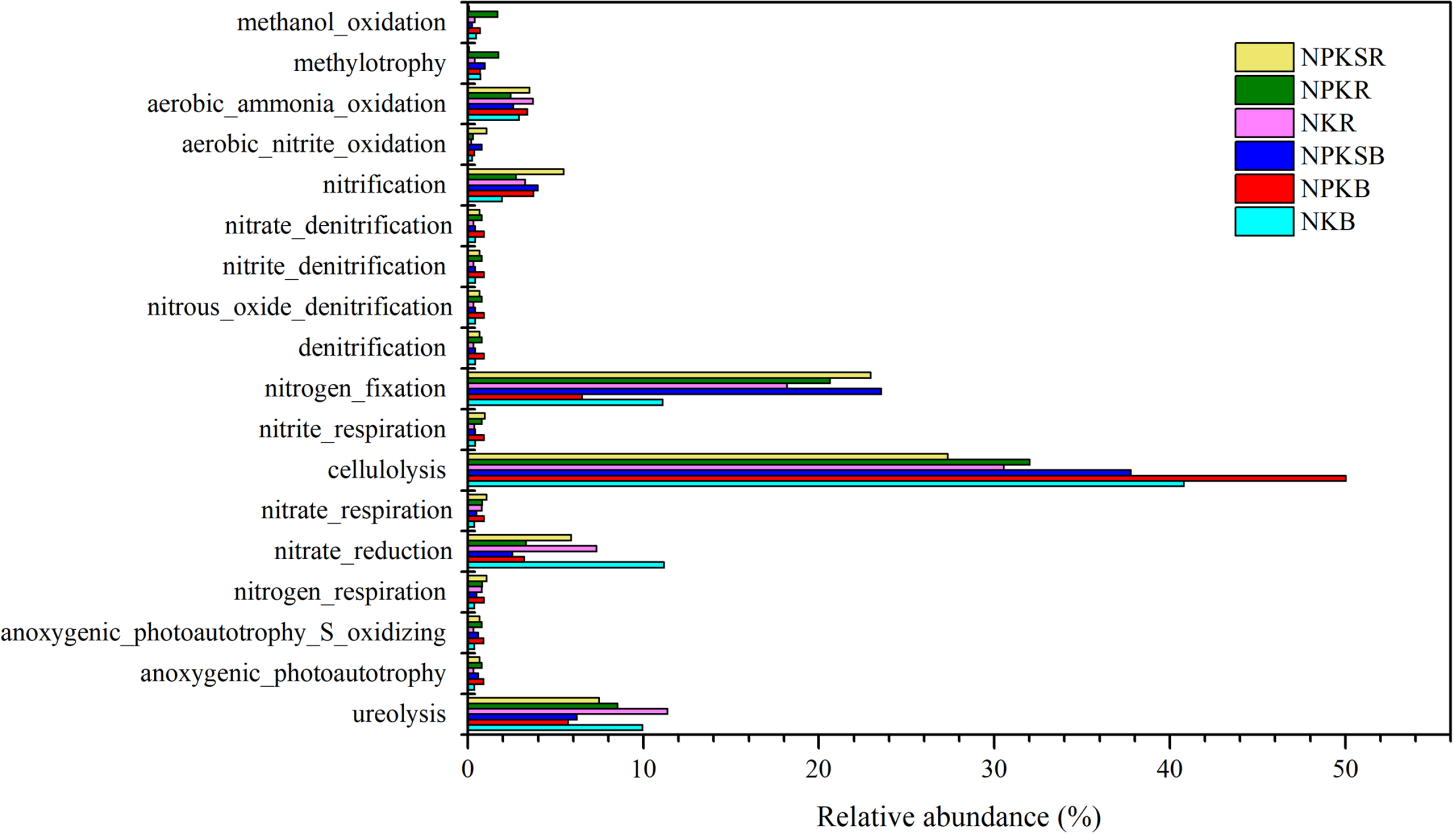
The relative abundances of putative microbial functions involved in C, N and S cycling under different fertilization treatments. NK, chemical nitrogen and potassium; NPK, chemical nitrogen, phosphorus and potassium; NPKS, NPK plus straw. B and R represent the BS and RS samples, respectively.

The potential functions of the bacteria communities were predicted by FAPROTAX (**Figure 8**). The results showed that those functional groups are mainly linked to biogeochemical carbon (C), N, and sulfur (S) metabolism. The functional groups involved in cellulolysis were significantly increased in NPK while reduced in NPKS in both BS and RS compared with NK (P<0.05). The relative abundances of functional groups methanol oxidation was increased in NPK and reduced in NPKS in both BS and RS, while functional groups methylotrophy were increased in NPK and reduced in NPKS only in RS. The P fertilization increased the relative abundances of functional groups anoxygenic_photoautotrophy_S_oxidizing and anoxygenic_photoautotrophy in both BS and RS.

N-cycling functional groups were also affected by different P fertilization treatments. The NPK and NPKS decreased the relative abundance of ureolysis and nitrate reduction functional groups in both BS and RS. The aerobic_nitrite_oxidation, nitrate_respiration and nitrogen_respiration groups increased in NPK and NPKS compared with NK in both BS and RS. The NPK and NPKS increased the relative abundances of functional groups nitrification only in BS, while increased the nitrogen_fixation only in RS. However, other functional groups such as nitrate_denitrification, nitrite_denitrification, denitrification, and nitrite_respiration were not significantly different among the three fertilization treatments.

## Discussion

Microorganisms are sensitive to agricultural practices such as fertilization and tillage, and they can adapt to new environments quickly (Dash et al., 2013; Ran et al., 2021). In the BS, after 7 years of fertilization, P fertilization simultaneously increased the bacterial richness (Chao1 index) and diversity (Shannon index), which supported our first hypothesis. Similarly, phosphate fertilizer addition has been found can increase the abundance of bacteria (16S rRNA gene copies) in an intercropped field (Tang et al., 2016). In the RS, in contrast to the BS, the bacterial Shannon index in the NPKS treatment was significantly lower than that in the NK treatment, while the observed species and richness were not influenced by P addition (**Supplementary Table 1**). Long-term P fertilization has also been found to significantly decrease the observed OTU number and bacterial diversity in wheat rhizosphere soils that have available P deficiencies (Liu et al., 2020). However, several studies have reported that P application does not significantly affect the bacterial observed OTUs and Shannon index (Ran et al., 2021) or bacterial community compositions (Li et al., 2019). Differences in these results may be due to the different rates and types of P fertilizer applications, soil conditions, and plant types (Samaddar et al., 2018; Li et al., 2019). Furthermore, our results revealed that the Shannon index was significantly higher in RS than in the BS, while there were no significant differences in the Chao1 or observed species indices between the BS and RS (**Figure 3**). Previous studies have reported lower bacterial Chao1 and Shannon indices in the RS than in the BS (Schlatter et al., 2020; Ran et al., 2021); there were no noticeable differences in soil bacterial ACE and Shannon indices between the RS and BS (Pang et al., 2021). Large differences in soil properties, organic compounds secreted by sugarcane, and the complex interaction of plant roots with soil can cause differences in soil microorganisms between RS and BS.

The soil bacterial compositions were also strongly affected by fertilizer P application, with more significant differences in the species in the RS than in the BS (**Figure 4**; **Supplementary Tables 2, 3**). This could be due to environmental factors that shaping the rhizosphere bacterial compositions was more complicated than those in the bulk soil (Schlatter et al., 2020). Similarly, it has been shown that the bacterial community shifts more in the RS than in the BS for wheat (Donn et al., 2015; Ran et al., 2021). At the phylum and genus levels, there were significant differences in the compositions between the NK and P addition (NPK and NPKS) treatments. For instance, compared with the NK treatment, the NPKS treatment significantly increased the relative abundances of Proteobacteria and Acidobacteria in the RS, whereas the NPK treatment decreased the abundance of Gemmatimonadetes in the RS. Proteobacteria, Acidobacteria and Gemmatimonadetes were the dominant phyla in the BS and RS of sugarcane. These abundant bacterial taxa have different life histories and functions, and dramatic changes in these phyla could influence soil nutrients and plant growth (Donn et al., 2015; Walters et al., 2018; Teng et al., 2018). Correlation analysis showed that Acidobacteria was significantly (P<0.05) positively correlated with all the agronomic and quality characteristics of sugarcane in our study (**Table 2**). At the genus level, the NPKS treatment significantly increased the abundances of *Bradyrhizobium*, *JG30-KF-AS9* and *Subgroup_2* but decreased that of *Conexibacter* in the RS and BS compared with the NK treatment. *Bradyrhizobium* and *Subgroup_2* was positively correlated with sugarcane yield, stalk height and stalk diameter, whereas *Conexibacter* showed the opposite trend. These results suggest that long-term P addition can alter the abundance of bacteria that affect nutrient cycling under low-P stress, which may alter the available nutrient supply and sugarcane growth. In fact, higher soil available N, available P and sugarcane yields were observed in the NPK and NPKS treatments than those in the NK treatment.

LEfSe analysis revealed that, compared with the bulk soil, a large number of bacterial genera were highly enriched in the sugarcane rhizosphere, mainly *Bradyrhizobium*, *Rhodanobacter*, *Pseudolabrys*, *Pseudaminobacter*, *1921-2*, *Burkholderia-Caballeronia-Paraburkholderia*, *Conexibacter*, *Sinomonas* and *Geodermatophilus*. Most of these sugarcane rhizosphere communities belong to a relatively small number of microbial taxa within the phyla Proteobacteria, Actinobacteria and Chloroflexi, consistent with previous findings (Donn et al., 2015; Schlatter et al., 2020). Yang et al. (2021) also have found that *Bradyrhizobium*, *1921-2* and *Conexibacter* were the dominant bacteria under different N fertilization treatments in sugarcane fields in China. *Bradyrhizobium* is a nitrogen fixer, and it has also been found to promote rock phosphate dissolution (Halder et al., 1990) and inhibit plant disease (Pang et al., 2021). The abundances of *Pseudolabrys* have been found increased in fertilization treatments that lacked P (Eo and Park, 2016). *Conexibacter* is believed to contribute to carbon cycling in soil environments and has the potential to reduce nitrate to nitrite (Deng et al., 2014). *Rhodanobacter* can reduce nitrate in the environment, and *Rhodanobacter* has been found to thrive under conditions of high nitrate and uranium contents and low pH (Green et al., 2012). Low pH values and high nitrate contents can cause selective pressure on the bacterial community, which explains the low bacterial diversity and high abundance of *Rhodanobacter* in the NPKSR (**Supplementary Table 1** and **Figure 5**). Above all, enrichment of *Bradyrhizobium*, *Conexibacter*, *Pseudolabrys* and *Rhodanobacter* in the sugarcane rhizosphere can affect the cycling of carbon, N and P. The bacteria *Sinomonas* and *Burkholderia-Caballeronia-Paraburkholderia* are plant growth promoters with the ability to provide phytohormones and important enzymes (Verbon and Liberman, 2016); the high abundance of these bacteria NKR can be explained by the beneficial plant-soil feedback under low-P stress conditions.

Changes in environmental factors, such as pH, organic carbon, and available N, can directly alter the soil microbial communities in different ecosystems (Pang et al., 2021; Zhao et al., 2021). In this study, RDA revealed that pH, SOM, TP, AP, AN and AK significantly influenced the soil bacterial communities in the BS and RS (**Figure 6**). Our results were consistent with previous findings in sugarcane, which showed that organic carbon, AP and pH determined soil bacterial dissimilarities (Pang et al., 2021). These studies suggested that soil AP and SOM were the dominant drivers of changes in the total bacterial community structure in the sugarcane field. Additionally, soil bacterial species also showed a close relationship with the soil P forms; for example, Proteobacteria and *Bradyrhizobium* were significantly and positively correlated with soluble Po, whereas *Conexibacter* and *1921-2* was significantly and negatively correlated with retained Po (**Figure 7**). Proteobacteria have been reported to express ACP and promote the mineralization of retained P, and *Bradyrhizobium* was significantly and positively correlated with soluble Po (Zhang et al., 2021). *Conexibacter* is believed to contribute to carbon cycling in soil ecosystems (Seki et al., 2012), which may simultaneously influence soil P cycling. Moreover, the soluble Po contents were higher in the NK treatment than NPK in both the RS and BS, which may be due to the enrichment of Proteobacteria in the NKR (Zhang et al., 2021) and the enrichment of *Arcticibacter*, a PSB, in the NKB. The retained Po contents were lower in the RS than in the BS in the NK and NPK treatments, likely due to *Conexibacter* enrichment in the NK treatment and *1921-2* enrichment in the NPK treatment in the sugarcane rhizosphere. These results suggested that variations in bacterial compositions can affect soil P cycling (Teng et al., 2018).

FAPROTAX can provide useful information on bacterial community functions and was widely used in soil research (Louca et al., 2016). The relative abundances of functional groups cellulolysis and methanol_oxidation were increased in NPK while reduced in NPKS in both BS and RS compared with NK, which might related to the higher proportion of *WPS-2* in NPK and the lower proportion of *WPS-2* in NPKS than that in NK (Yang et al., 2021) (**Supplementary Table 3**). The relative abundance of functional groups anoxygenic_photoautotrophy_S_oxidizing and anoxygenic_photoautotrophy increased with NPK and NPKS might be dependent on the high proportion of Cyanobacteria in these two treatments. The NPK and NPKS treatments decreased the relative abundance of ureolysis and nitrate reduction functional groups compared with NK, which might due to the lower abundance of *Conexibacter* in P fertilization treatments than that in NK (Deng et al., 2014). However, further work is needed to compare the different roles of fertilization treatments on bacterial community function between BS and RS.

## Conclusions

After 7 years of the same fertilizer regime, P application significantly increased sugarcane yield and quality characteristics compared with those in the NK treatment, and soil TP, soluble Pi and retained Pi in both the RS and BS increased. Although the main phyla and genera of the bacterial communities were similar between the BS and RS, the compositions and abundances of the bacteria were significantly different and affected by P fertilization, with more obvious changes in the RS than in the BS. A significant number of bacterial genera were enriched in the sugarcane rhizosphere, most of which are potential benefit for the plant growth. The P fertilization treatments also significantly increased or decreased the functional groups involved in the cycling of C, N, and S compared with NK. Additionally, many bacterial species were significantly correlated with soil P forms, indicating that soil bacteria played an important role in soil P cycling. These results revealed the different effects of long-term P fertilization on soil nutrients and bacterial communities in sugarcane soil under low-P stress. They also provided new insights to stimulate the power of the microbes by agricultural measures to improve soil nutrients and crop production.

## Data availability statement

The original contributions presented in the study are publicly available. This data can be found here: https://doi.org/10.6084/m9.figshare.20500110.v3.

## Author contributions

QW: conceptualization, methodology, writing–original draft, writing–review and editing, and funding acquisition. DC, WZ: methodology, formal analysis, and data curation. XZ: conceptualization, methodology. JA: conceptualization, supervision, and funding acquisition. All authors contributed to the article and approved the submitted version.

## Funding

This work is supported by the GDAS Special Project of Science and Technology Development [2019GDASYL-0103033, 2020GDASYL-20200103059], the National Key R&D Program of China [2020YFD1000600] and the China Agricultural Research System of MOF and MARA [CARS-170203].

## Conflict of interest

The authors declare that the research was conducted in the absence of any commercial or financial relationships that could be construed as a potential conflict of interest.

## Publisher’s note

All claims expressed in this article are solely those of the authors and do not necessarily represent those of their affiliated organizations, or those of the publisher, the editors and the reviewers. Any product that may be evaluated in this article, or claim that may be made by its manufacturer, is not guaranteed or endorsed by the publisher.
