# Supplementary figures and images for: Long-term fertilization has different impacts on bacterial communities and phosphorus forms in sugarcane rhizosphere and bulk soils under low-P stress
Source: Front Plant Sci. 2022 Sep 23;13:1019042. doi: 10.3389/fpls.2022.1019042 (PMC9539793; doi:10.3389/fpls.2022.1019042)

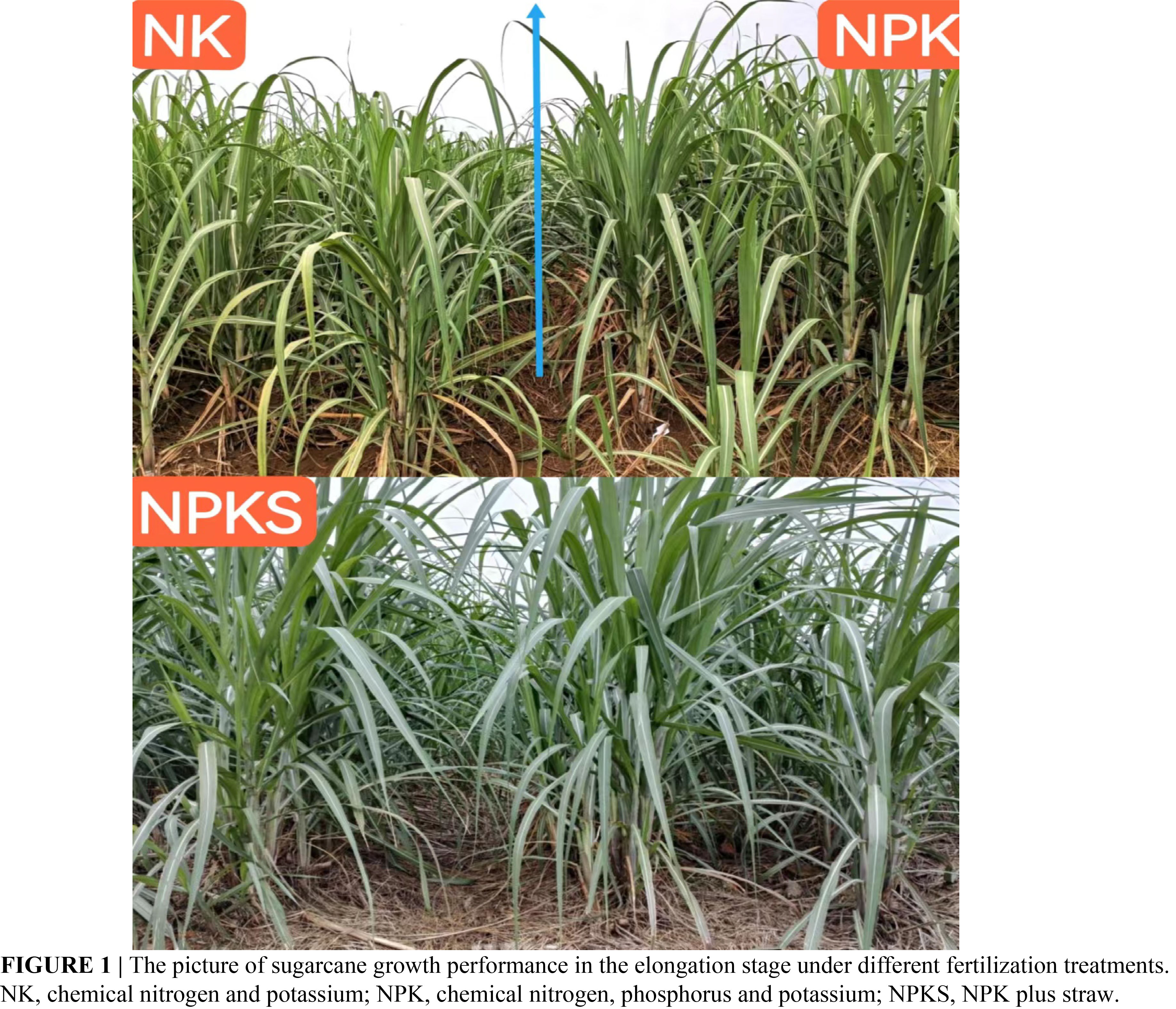

Supplement: Supplementary file 1 [file Image_1.jpeg]

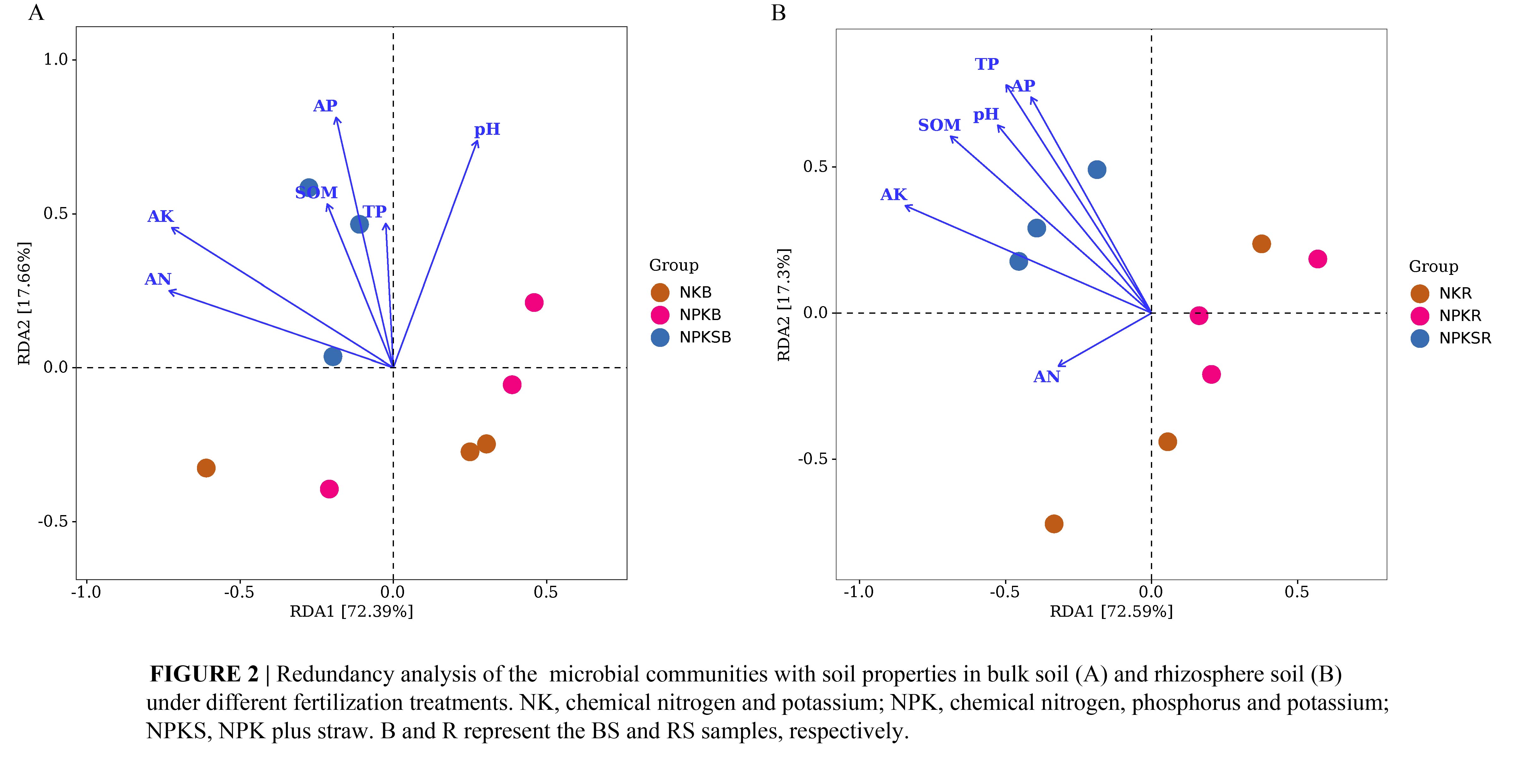

Supplement: Supplementary file 2 [file Image_2.jpeg]
