# Supplementary material for: Long-term fertilization has different impacts on bacterial communities and phosphorus forms in sugarcane rhizosphere and bulk soils under low-P stress
Source: Front Plant Sci. 2022 Sep 23;13:1019042. doi: 10.3389/fpls.2022.1019042 (PMC9539793; doi:10.3389/fpls.2022.1019042)
Supplement: Supplementary file 3 [file DataSheet_1.docx]

**Table 1** Bacterial diversity indices in the different fertilization treatments.

| Treatment | BS | | |  | RS | | |
| --- | --- | --- | --- | --- | --- | --- | --- |
|  | Observed species | Chao1 | Shannon |  | Observed species | Chao1 | Shannon |
| NK | 3129Ab | 3488Bb | 9.26Bb |  | 3696Aa | 4079Aa | 10.00Ab |
| NPK | 3603Aab | 4006Aa | 9.68Ba |  | 3989Aa | 4409Aa | 10.30Aa |
| NKPS | 4010Aa | 4259Aa | 9.95Aa |  | 3891Aa | 4046Aa | 9.79Bc |

NK, chemical nitrogen and potassium; NPK, chemical nitrogen, phosphorus and potassium; NPKS, NPK plus straw. Different lowercase letters in the same column indicate significant differences among the three P treatments (*P*<0.05). Different uppercase letters in the same row indicate significant differences for each index between the bulk soil (BS) and rhizosphere (RS).

**Table 2** The relative abundances of the 11 most abundant phyla in the different fertilization treatments.

| Taxon | Relative abundances (%) in BS | | | Relative abundances (%) in RS | | |
| --- | --- | --- | --- | --- | --- | --- |
|  | NK | NPK | NPKS | NK | NPK | NPKS |
| Chloroflexi | 21.1a | 29.0a | 13.8a | **21.3ab** | **27.8a** | **11.7b** |
| Proteobacteria | 34.0a | 33.4 a | 40.3a | **31.5b** | **28.3b** | **40.7a** |
| Acidobacteria | **6.1b** | **8.9ab** | **14.6a** | **7.1b** | **9.5ab** | **13.5a** |
| Actinobacteria | 25.7a | 19.8a | 20.6a | 28.7a | 22.4a | 25.1a |
| WPS-2 | 3.7a | 3.4a | 1.6a | **3.7b** | **5.9a** | **1.4c** |
| Planctomycetes | 1.3a | 1.3a | 1.0a | 0.9a | 1.4a | 0.9a |
| Verrucomicrobia | **0.3b** | **0.4b** | **0.9a** | 0.3a | 0.4a | 0.7a |
| Bacteroidetes | 2.2a | 1.0a | 0.7a | 1.8a | 1.3a | 0.8a |
| Patescibacteria | 0.6a | 0.6a | 0.5a | 1.0a | 0.5a | 0.5a |
| Firmicutes | 1.4a | 0.6a | 1.2a | 1.4a | 0.5a | 1.5a |
| Gemmatimonadetes | 2.1a | 0.5a | 3.8a | **1.4ab** | **0.6b** | **2.3a** |

NK, chemical nitrogen and potassium; NPK, chemical nitrogen, phosphorus and potassium; NPKS, NPK plus straw. Different lowercase letters in the same row indicate significant differences among the three P treatments in the bulk soil (BS) or rhizosphere (RS) (*P*<0.05).

**Table 3** The relative abundances of the 16 most abundant genera in the different fertilization treatments.

| Taxon | Relative abundances (%) in BS | | | | Relative abundances (%) in RS | | |
| --- | --- | --- | --- | --- | --- | --- | --- |
|  | NK | NPK | NPKS | NK | | NPK | NPKS |
| *Chujaibacter* | 10.8a | 10.3a | 4.5a | 5.8a | | 2.6a | 5.4a |
| *Acidothermus* | 6.7 a | 4.6a | 5.8a | 7.6a | | 5.4a | 4.4a |
| *Bradyrhizobium* | **1.3b** | **1.5b** | **3.6a** | **2.0b** | | **2.5b** | **3.7a** |
| *Bryobacter* | 1.4a | 1.4a | 2.0a | 2.1a | | 2.0a | 2.6a |
| *Subgroup_2* | **0.6b** | **1.3b** | **3.2a** | **0.4b** | | **0.8b** | **2.5a** |
| *JG30-KF-AS9* | **0.6b** | **1.0b** | **2.7a** | **0.9b** | | **0.9b** | **2.4a** |
| *Acidibacter* | 1.2a | 1.8a | 1.8a | 1.3a | | 1.1a | 2.3a |
| *Actinospica* | 2.7a | 2.3a | 1.7a | 2.7a | | 2.4a | 2.1a |
| *HSB_OF53-F07* | 1.4a | 2.2a | 2.0a | 1.2a | | 2.3a | 1.7a |
| *FCPS473* | 1.3a | 2.0a | 1.6a | **2.3ab** | | **3.0a** | **1.6b** |
| *WPS-2* | 3.7a | 3.4a | 1.6a | **3.7b** | | **5.9a** | **1.4c** |
| *Conexibacter* | **3.4a** | **2.6ab** | **0.9b** | **3.6a** | | **3.3a** | **1.3b** |
| *AD3* | 10.9a | 15.9a | 2.3a | 9.2a | | 10.2a | 1.2a |
| *Sphingomonas* | 1.1a | 1.2a | 1.0a | 1.9a | | 2.7a | 1.1a |
| *IMCC26256* | 1.1a | 1.2a | 0.8a | 1.2a | | 1.5a | 0.8a |
| *1921-2* | 1.4a | 1.8a | 0.4a | **1.1b** | | **2.6a** | **0.2c** |

NK, chemical nitrogen and potassium; NPK, chemical nitrogen, phosphorus and potassium; NPKS, NPK plus straw. Different lowercase letters in the same row indicate significant differences among the three P treatments in the bulk soil (BS) or rhizosphere (RS) (*P*<0.05).
